# Sexual Well-Being in Patients with Blepharospasm, Spasmodic Torticollis, and Hemifacial Spasm: A Pilot Study

**DOI:** 10.3389/fpsyg.2016.01492

**Published:** 2016-10-05

**Authors:** Paola Perozzo, Adriana Salatino, Paolo Cerrato, Raffaella Ricci

**Affiliations:** ^1^Fondazione Carlo Molo Onlus, TurinItaly; ^2^SAMBA (SpAtial, Motor & Bodily Awareness) Research Group, Psychology Department, University of Turin, TurinItaly; ^3^Stroke Unit, Department of Neuroscience, University of Turin, TurinItaly; ^4^Neuroscience Institute of Turin, University of Turin, TurinItaly

**Keywords:** dystonia, hemifacial spasm, sexual function, depression, anxiety

## Abstract

Mood, anxiety, and other psychological symptoms are common in dystonic patients suffering from blepharospasm (BSP) and spasmodic torticollis (ST). Since sexual well-being is an important aspect of mental health, here, we investigated whether these patients may also experience a worsening of their sexual life. In particular, quality of sexual life was evaluated in patients suffering from BSP (*N* = 30), ST (*N* = 30), and in a control group of patient with Hemifacial spasm (HFS; *N* = 30), undergoing botulinum toxin type A therapy. A group of 30 age-matched healthy volunteers constituted an additional control group. Patients were evaluated just before the periodic injection of botulinum toxin. Sexual functioning was assessed using the Sexual Functioning Inventory, a reduced form of the Golombok Rust Inventory, previously employed in patients with Parkinson’s disease. Depression (Beck Depression Inventory) and anxiety (STAI-X1/X2) were also assessed. Results revealed that sexual functioning was significantly affected in patients with BSP, ST, and HFS with respect to healthy controls. Dystonic patients manifested more sexual dysfunction than patients with HFS. Overall, females had a poorer quality of sexual life than males and, among females, women with BSP were the most dysfunctional. Psychological symptoms were present in patients with dystonia, but not in patients with HFS. As discussed in the paper, several factors might be taken into account to explain worse quality of sexual life in patients with dystonia compared to patients with hemifacial spasm. Among them an important role might be played by the central origin of dystonia pathophysiology (i.e., altered activity of cortico-striato-thalamic-cortical circuits). Future investigations are necessary to further explore these preliminary findings, considering that this is the first time that sexual well-being is evaluated in patients with BSP, ST, and HFS, and comparable data are not available.

## Introduction

Dystonia is a neurological movement disorder in which sustained or intermittent muscle contractions result in abnormal, often repetitive movements, and/or postures. Blepharospasm (BSP) and spasmodic torticollis (ST) are two of the most common forms of dystonia (Albanese et al., 2013). BSP is a focal dystonia characterized by bilateral involuntary contraction of orbicularis oculi muscles with the consequence of continuous or intermittent forceful closure of the eyelids (Dashtipour et al., 2015). Neural circuits underlying the pathophysiology of BSP involve the basal ganglia, the thalamus, and the peduncle-mesencephalic area (Defazio et al., 2007). ST is the most common form of idiopathic focal dystonia and is characterized by intermittent or sustained deviation of the neck, accompanied by pain. The pathophysiology of ST seems to specifically involve the basal ganglia and their connections to cortical areas (Jinnah et al., 2013; Avanzino and Fiorio, 2014).

Recent studies report the presence of mood, anxiety, and other psychological symptoms in patients with BSP and ST, and suggest that successful local treatment of movement disorders, typically done with botulinum toxin therapy (BoNTA), is not necessarily linked to patients’ improvement in social ability and emotional state (Tucha et al., 2001; Reimer et al., 2005; Hall et al., 2006; Slawek et al., 2007; Setthawatcharawanich et al., 2011; Stamelou et al., 2012; Berardelli et al., 2015). In these studies, BSP is very often compared to Hemifacial spasm (HFS), that, by being a peripherally induced movement disorder, might be considered a natural control condition. HFS is characterized by clonic-tonic contractions of the orbicularis oculi muscles, usually of one-half of the face, and is typically caused by facial nerve irritation due to a neurovascular conflict (Wang and Jankovic, 1998). Despite improvement of dystonic manifestations due to the treatment, patients with BSP show higher levels of depression, anxiety, and obsessive-compulsive symptoms and, in general, a worse quality of life, than patients with HFS (Scheidt et al., 1996; Broocks et al., 1998; Tucha et al., 2001; Tan et al., 2005, 2006; Hall et al., 2006; Barahona-Corrêa et al., 2011; Fontenelle et al., 2011; Setthawatcharawanich et al., 2011; Lehn et al., 2014). Nonetheless, few authors do not report differences in obsessive-compulsive symptoms between the two diseases (Hall et al., 2005; Munhoz et al., 2005; Fontenelle et al., 2011). The findings of worse psychological symptoms in BSP than HFS might be related to the presence of more severe visual impairment and ophthalmological pain (Martino et al., 2005) with consequences on everyday life (Reimer et al., 2005; Hall et al., 2006). However, it might also be ascribed to dysfunction of the cortico-subcortical circuits (i.e., cortico-striatal circuitry comprising fronto-orbital and cingulate structures) that are typically involved in mood disorders (Broocks et al., 1998; Munhoz et al., 2005; Reimer et al., 2005; Barahona-Corrêa et al., 2011; Conte et al., 2016). Studies investigating psychological variables in patients with ST, undergoing BoNTA treatment, report that the most common psychological symptoms manifested by these patients are anxiety (Wenzel et al., 1998; Gündel et al., 2003), depression (Jahanshahi and Marsden, 1988; Pekmezovic et al., 2009; Fabbrini et al., 2010), and social embarrassment accompanied by social avoidance (Jahanshahi, 1991; Comella and Bhatia, 2015). One study (Gündel et al., 2001), evaluating 116 patients with ST, shows that more than 50% of them fulfilled modified DSM-IV criteria for social phobia, independently of the severity of the disorder. Moreover, some authors consider depression a consequence of postural abnormalities (Jahanshahi and Marsden, 1990, 1992), while others as an independent factor (Stamelou et al., 2012) and the main predictor of worse quality of life (Slawek et al., 2007). An interesting study reports that the majority of ST patients perceived some or severe stigma mainly due to physical disfigurement (Papathanasiou et al., 2001).

Thus, there is evidence of mood, anxiety, and other psychological symptoms in patients with BSP and ST, undergoing BoNTA treatment, but none of the above studies has investigated whether these patients may also experience a worsening of their sexual life. Since sexual functioning is an important aspect of mental health (World Association for Sexual Health [WAS], 2008), and it has been found to be deficient in neurological patients with other types of movement disorders (such as, for example Parkinson’s disease, Castelli et al., 2004), in the current study, we investigated whether dystonic patients with BSP and ST may also experience a worsening of their sexual life. We also explored the putative relationship of this aspect of mental health with other psychological factors often aﬄicting patients with dystonia.

To these aims, we assessed the presence of psychological symptoms (i.e., anxiety and depression) and of sexual dysfunction in male and female patients with BSP and ST and in a control group of patients with HFS. Another control group was constituted by age-matched healthy controls. Sexual functioning was evaluated through the Sexual Functioning Inventory (SFI), a tool that proved to be sensitive in the assessment of sexual dysfunction in neurological patients with Parkinson’s disease (Castelli et al., 2004). The existence of possible correlations between sexual functioning, the above psychological aspects, or gender and age was also evaluated.

On the basis of previous findings, we expected to observe more severe psychological symptoms in patients with dystonia (BSP and ST) than in patients with HFS. In addition, we predicted that if a relationship existed between psychological symptoms and sexual functioning, then worse quality of sexual life might also be expected in patients with dystonia compared to patients with HFS.

## Materials and Methods

### Participants

Ninety patients were submitted to a psychological assessment, at the San Giovanni Battista Hospital in Turin, Italy, before undergoing the periodic injection of botulinum toxin type A (BoNTA) therapy. Thirty of them were affected by BSP, 30 suffered from ST and 30 manifested HFS. In order to assess sexual well-being in patients with homogeneous and stable clinical conditions, only chronic patients (duration of illness ranged from 12 to 15 years), undergoing the BoNTA treatment for at least 8 years (the duration of the BoNTA treatment ranged from 8 to 11 years) were enrolled in the study. Most importantly, only patients with a moderate (grade 3: functional impairment) disability (Jankovic et al., 1990) were included in the study. The exclusion criteria from the study were the presence of dementia, psychiatric disease such as Psychosis or Major Depression, motor disorder like Parkinson’s disease or other type of comorbidities and the presence of autonomic urogenital dysfunction on interview or physical examination. None of the patients had history of alcohol or drug abuse.

A healthy control group of 30 participants was matched to patients for sex, age, and educational level. Patients and control participants were all heterosexual. In **Table 1** are reported demographic and clinical data for each group of participants.

**Table 1:** Demographic and clinical characteristics.

Group	Sex	Age	Education
Blepharospasm (BSP)	M (15)	66.20 (4.46)	5.93 (2.52)
	F (15)	66.33 (10.51)	6.60 (1.55)
Hemifacial spasm (HFS)	M (15)	64.00 (11.59)	8.07 (3.01)
	F (15)	67.33 (6.04)	7.73 (3.33)
Spasmodic torticollis (ST)	M (15)	61.53 (8.63)	8.73 (2.91)
	F (15)	67.77 (6.44)	7.40 (2.67)
Controls	M (15)	63.27 (2.12)	7.47 (2.67)
	F (15)	63.20 (2.14)	7.53 (2.67)

The study was carried out in accordance with the recommendations of the Declaration of Helsinki. Participants were given a detailed explanation of the study and an informed consent, previously approved by the Local Ethical Committee of the hospital, was obtained from all individuals included in the study.

### Psychological Assessment

Depression was evaluated using the Beck Depression Inventory (BDI; Beck, 1987), a 21-item self-rating scale investigating cognitive, behavioral, and somatic aspects of depression.

Anxiety was assessed using the State-Trait Anxiety Inventory (STAI; Spielberg et al., 1980), a 40-item self-rating scale assessing anxiety as a reaction to episodic stress condition (STAI-X1) or as a general predisposition for anxious behavior (STAI-X2).

### Sexual Function Assessment

Sexual well-being was evaluated using the SFI (Castelli et al., 2004), a reduced form of the Golombok-Rust Inventory of Sexual Satisfaction (Rust and Golombok, 1986). SFI is a 15-item self-rating scale investigating seven sexual disorders: infrequency, non-communication, dissatisfaction, avoidance, non-sensuality, premature ejaculation-vaginismus, and impotence-anorgasmia. Two different forms of the questionnaire were used for men and women. The score ranges from 15 to 60 with higher scores indexing greater sexual dysfunction.

### Statistical Analysis

Differences between the three groups of patients and controls on SFI total score and each subscale were analyzed by means of ANOVAs with GROUP (BSP, HFS, ST, and Controls) and GENDER (Males and Females) as between-subjects factors. Data analysis was also performed including the BDI and the STAI as covariates (ANCOVA) in order to evaluate their role on sexual well-being.

Differences between the three groups of patients and controls on the BDI, STAI-X1, and STAI-X2, were also investigated by means of ANOVAs with GROUP (BSP, HFS, ST, and Controls) and GENDER as between-subjects factors. *Post hoc* comparisons were performed using Tuckey’s HSD procedure.

The Pearson correlation coefficient was employed to assess correlations between sexual functioning scores, psychological tests, and socio-demographic variables (age and education) in patients.

## Results

No significant differences were found among the three groups of patients with respect to clinical and demographic characteristics. Patients did not differ from controls in demographic variables (age and education).

### Psychological Scales

Significant differences between patients and controls and among the groups of patients were found on the psychological scales (**Table 2**). The ANOVAs revealed a main effect of the factor GROUP for BDI [*F*(3,112) = 9.845, *p* < 0.001, $\eta_{p}^{2}$ = 0.209], STAI-X1 [*F*(3,112) = 10.476, *p* < 0.001, $\eta_{p}^{2}$ = 0.219], STAI-X2 [*F*(3,112) = 10.702, *p* < 0.001, $\eta_{p}^{2}$ = 0.223]. Tuckey’s HSD tests showed that patients with BSP and ST had significantly higher scores than patients with HFS and healthy participants on BDI, STAI-X1, and STAI-X2 (*p* < 0.006 for each comparisons). Moreover, HFS patients did not show any significant difference with healthy participants on these scales. To summarize, dystonic patients with BSP and ST scored worse on the psychological scales than patients with HFS. In addition, these patients had scores not significantly different from those of healthy individuals.

**Table 2:** Results of BDI, STAI-X1, and STAI-X2.

	BSP	HFS	ST	C
Beck Depression Inventory (BDI)	11.03 (6.24)	6.43 (3.14)	11.17 (7.88)	5.50 (0.86)
State-Trait Anxiety Inventory (STAI)-X1	41.17 (8.42)	34.97 (4.4)	40.07 (9.77)	32.63 (2.20)
STAI-X2	41.20 (7.12)	33.83 (5.94)	41.00 (10.79)	33.53 (2.73)

### Sexual Functioning

**Table 3** reports mean and standard deviations of the SFI scores for patients and controls.

**Table 3:** Sexual Functioning Inventory (SFI) results.

	BSP	HFS	ST	Controls
Infrequency	6.33 (0.84)	5.77 (1.28)	6.27 (1.08)	5.23 (0.73)
Non-communication	5.03 (1.83)	5.07 (1.72)	5.20 (1.32)	4.23 (1.19)
Dissatisfaction	6.40 (1.30)	5.77 (1.57)	6.10 (1.54)	4.93 (0.87)
Avoidance	4.17 (1.44)	3.87 (1.22)	4.17 (1.26)	3.47 (1.25)
Non-sensuality	2.83 (0.91)	2.77 (1.04)	3.10 (1.40)	2.20 (0.61)
Premature ejaculation/Vaginismus	2.77 (0.82)	2.47 (1.01)	2.37 (0.96)	2.00 (0.74)
Impotence/Anorgasmia	2.13 (1.07)	1.93 (0.91)	2.03 (0.56)	1.37 (0.56)
**Total score**	**31.80 (5.01)**	**29.60 (6.23)**	**31.30 (4.17)**	**25.63 (3.09)**

On the total SFI score, the ANOVA revealed significant main effects of GROUP [*F*(3,112) = 12.524, *p* < 0.0001, $\eta_{p}^{2}$ = 0.251] and GENDER [*F*(1,112) = 21.139, *p* < 0.001, $\eta_{p}^{2}$ = 0.159]. Tuckey’s HSD tests showed that BSP, HFS, and ST patients had a total SFI score significantly higher than healthy participants (*p* < 0.004 for each comparisons). Moreover, females had higher scores than males. Thus, on this test, the three groups of patients showed worse sexual functioning than healthy matched controls, and overall women scored worse than men. The ANOVAs performed on each subscale revealed that the main factor GROUP was significant for the following item: infrequency [*F*(3,112) = 8.237, *p* < 0.0001, $\eta_{p}^{2}$ = 0.181], dissatisfaction [*F*(3,112) = 6.759, *p* < 0.001, $\eta_{p}^{2}$ = 0.153], non-sensuality [*F*(3,112) = 3.997, *p* < 0.01, $\eta_{p}^{2}$ = 0.097], premature ejaculation-vaginismus [*F*(3,112) = 4.131, *p* < 0.01, $\eta_{p}^{2}$ = 0.100], and impotence-anorgasmia [*F*(3,112) = 7.689, *p* < 0.0001, $\eta_{p}^{2}$ = 0.171]. According to *post hoc* analyses, BSP and ST patients had higher scores than controls on the infrequency and dissatisfaction subscales, while HFS patients did not differ from controls on these subscales (*p* < 0.006 for each comparisons). In addition, BSP patients had higher scores than controls on the premature ejaculation-vaginismus subscale and ST patients on the non-sensuality subscale (*p* < 0.007 for each comparisons). The three groups of patients showed more disorders of impotence-anorgasmia than controls (*p* < 0.007 for each comparisons). The ANOVAs also revealed a significant main effect of GENDER on the following subscales: infrequency [*F*(1,112) = 7.875, *p* < 0.01, $\eta_{p}^{2}$ = 0.066], non-communication [*F*(1,112) = 18.370, *p* < 0.001, $\eta_{p}^{2}$ = 0.141], avoidance [*F*(1,112) = 49.790, *p* < 0.0001, $\eta_{p}^{2}$ = 0.308], premature ejaculation-vaginismus [*F*(1,112) = 13.266, *p* < 0.001, $\eta_{p}^{2}$ = 0.106], and impotence-anorgasmia [*F*(1,112) = 41.782, *p* < 0.001, $\eta_{p}^{2}$ = 0.272]. Females had higher scores than males on all the above subscales except for the premature ejaculation-vaginismus item, where men scored worse than women (**Table 4**). These findings were not affected by the inclusion of BDI and STAI as covariates in the analyses, suggesting that these variables did not affect the above results.

**Table 4:** Results of the SFI for females and males.

	Male	Female
Infrequency	5.65 (1.19)	5.65 (1.19)
Non-communication	4.32 (1.35)	5.45 (1.58)
Dissatisfaction	5.68 (1.44)	5.92 (1.44)
Avoidance	3.22 (0.74)	4.62 (1.39)
Non-sensuality	2.77 (1.16)	2.68 (0.98)
Premature ejaculation/Vaginismus	2.68 (0.85)	2.12 (0.90)
Impotence/Anorgasmia	1.47 (0.60)	2.27 (0.88)
**Total score**	**27.77 (4.60)**	**31.40 (5.36)**

A significant interaction GROUP X GENDER was found on the impotence-anorgasmia subscale [*F*(3,112) = 3.337, *p* < 0.05, $\eta_{p}^{2}$ = 0.082]. Tuckey’s HSD tests showed that BSP females had a significantly higher score (*M* = 2.87, *SD* = 0.99) than BSP males (*M* = 1.40, *SD* = 0.51), ST males (*M* = 1.73, *SD* = 0.46), HFS males (*M* = 1.60, *SD* = 0.83), control males (*M* = 1.13, *SD* = 0.35), and control females (*M* = 1.60, *SD* = 0.63; *p* < 0.001 for each comparisons); ST females had significantly higher score (*M* = 2.33, *SD* = 0.49) than BSP males and control males (*p* ≤ 0.01 for each comparisons); similarly, HFS females had significantly higher score (*M* = 2.27, *SD* = 0.88) than BSP males and control males (*p* < 0.007 for each comparisons).

### Correlations

Pearson analyses revealed that BDI positively correlated with the SFI total score (*r* = 0.238, *p* = 0.024), and the infrequency subscale (*r* = 0.287, *p* = 0.006). STAI-X1 (*r* = 0.306, *p* = 0.003) and STAI-X2 (*r* = 0.320, *p* = 0.002) scores positively correlated with infrequency subscale.

Positive correlations were also found between age and total SFI score (*r* = 0.293, *p* = 0.005), infrequency (*r* = 0.296, *p* = 0.005), non-communication (*r* = 0.320, *p* = 0.002), premature ejaculation-vaginismus (*r* = 0.232, *p* = 0.028), and impotence-anorgasmia (*r* = 0.318, *p* = 0.002) subscales.

## Discussion

There is evidence of mood and anxiety symptoms in dystonic patients with blepharospasm and (ST) undergoing BoNTA therapy, but no data are available on their sexual well-being. Here, we assessed these patients’ sexual functioning and its putative relationship with other aspects of mental health (i.e., depression and/or anxiety). To this aim, patients with a peripherally induced movement disorder, i.e., HFS, and age-matched healthy volunteers constituted the control groups.

The results of the present study showed the presence of depressive and anxious symptoms in patients with BSP and ST, but not in patients with HFS. Overall, these findings are in line with the evidence of higher levels of depression and anxiety, obsessive compulsive, and social phobia symptoms, associated with poorer quality of life in BSP and ST compared to HFS patients (Bihari et al., 1992; Hall et al., 2006; Tan et al., 2006; Pekmezovic et al., 2009; Barahona-Corrêa et al., 2011; Fontenelle et al., 2011; Setthawatcharawanich et al., 2011; Lehn et al., 2014). Most importantly, although sexual functioning was affected in all groups of patients (i.e., higher incidence of impotence in men and anorgasmia in women), dystonic patients were the most compromised. Men and women with BSP reported additional physiological dysfunctions (i.e., premature ejaculation and vaginismus, respectively), that likely affected the frequency of intercourse events. Infrequency of intercourse was also reported by ST patients, who also showed lower scores on non-sensuality (i.e., enjoying cuddling and caressing) subscale. This latter outcome might be related to embarrassment caused by postural abnormalities and perception of physical disfigurement. The degree of sexual dysfunction of the two groups of dystonic patients was reflected in their level of dissatisfaction: both groups were not satisfied with their sexual life, while HFS patients reported the same level of satisfaction as healthy controls. Finally, all groups of patients had normal scores on the sexual avoidance subscale, suggesting that interpersonal and emotional aspects of sexual life were overall preserved, especially in patients whose movement disorders were confined to craniofacial muscles.

Besides the type of neurological disease, another factor affecting sexual functioning was gender. Overall women had a poorer quality of sexual life than men in relation to several psychological components (i.e., frequency of intercourse events, communication, and avoidance) and one physiological aspect (i.e., on the impotence/anorgasmia item). Men scored worse than women only on the premature ejaculation-vaginismus item. Overall, women with BSP resulted to be the most compromised on the anorgasmia item.

Correlation analyses showed that depression as well as age were positively related to sexual dysfunction (i.e., total SFI score). Indeed, both factors are crucial determinants of sexual well-being (Lipe et al., 1990; Welsh et al., 1997; Moore et al., 2002; Bronner et al., 2014). Importantly, our findings revealed that higher levels of depression and anxiety were specifically related to less frequent intercourse events. On the other hand, age correlated with the number of physiological dysfunctions, and negatively affected the frequency of intercourse and communication on sexual life. These results are in line with previous findings (Gott and Hinchliff, 2003; Ni Lochlainn and Kenny, 2013) showing that worsening of sexual life with age is especially due to physiological factors and difficulties in talking about sexual issues with the partner and/or the specialist.

Since, to our knowledge, this is the first study on sexual well-being in BSP, ST, and HFS patients, comparable data are not available. However, our findings are consistent with previous evidence showing sexual dysfunction in patients affected by another type of movement disorder, i.e., Parkinson’s disease (Lambert and Waters, 1998; Bronner et al., 2014), and its improvement, following deep brain stimulation of the subthalamic nucleus, in men but not in women (Castelli et al., 2004). Moreover, they are in line with the evidence that quality of life is poorer in women than in men with BSP (Müller et al., 2002).

To summarize, our findings show the presence of depressive and anxiety symptoms and worse quality of sexual life in patients with dystonia compared to patients with HFS and age-matched healthy subjects. This outcome and the presence of positive correlation between depression/anxiety and sexual dysfunction suggest that psychological symptoms and sexual well-being might be related in these patients. Several factors might be taken into account to explain more severe psychological symptoms in dystonic patients. Some authors underline the presence of severe visual impairment, ophthalmological pain, and driving difficulty in BSP (Martino et al., 2005; Reimer et al., 2005; Hall et al., 2006). Pain is an important variable that significantly affects general well-being also in ST (Chan et al., 1991; Slawek et al., 2007; Comella and Bhatia, 2015), and neck pain is a main determinant of depression in these patients (Müller et al., 2002). Another aspect, that might account for psychological symptoms in dystonic patients is self-consciousness of the altered physical appearance. A previous study (Papathanasiou et al., 2001) underlines that social stigma in ST patients, mainly related to physical appearance, negatively affects interpersonal relationships and, we hypothesize, sexual well-being too. Other authors (Jahanshahi and Marsden, 1990) found that negative body image has an important impact on depression and psychological well-being in ST. Besides pain and negative body concept due to disfigurement, psychological symptoms might be, at least in part, related to the pathophysiology of dystonic syndromes. Indeed, dysfunction of cortico-basal ganglia circuits may be associated with mood and anxiety disorders (Marchand, 2010). In addition, the central origin of dystonia pathophysiology, i.e., altered activity of cortico-striato-thalamic-cortical circuits (Quartarone and Hallett, 2013), might also have a role in sexual dysfunction. On the one hand, reduction of striatal dopamine leads to disinhibition of fronto-orbital and cingulate cortices, that are involved in regulation of motivation and emotion. On the other hand, chronic anxiety might induce a dysfunctional state of hypocortisolism and further reduce dopamine levels in the striatum (Pruessner et al., 2004; Hall et al., 2005; Defazio et al., 2007; Barahona-Corrêa et al., 2011), that have a key role in both sexual arousal and desire (Giuliano and Allard, 2001; Brom et al., 2014).

Despite sexual well-being might be a consequence of psychological factors and/or altered activity of cortico-subcortical circuits, the correlational nature of our data do not allow to draw conclusions about the causal role of the above factors on sexual dysfunction. Another limitation of the current study is the absence of a measure of the patients’ global quality of life. Because of outpatients’ time constraints, we had to employed a limited number of clinical tests. The use of additional clinical evaluations (for example the SF-36 scale), besides SFI, BDI, and STAI, would have allowed to correlate our findings to other measures of the patients well-being in order to draw clearer and stronger conclusions. Future studies employing more extensive clinical examination and, when possible, neurophysiological, or neuroimaging measures of brain activity, in larger groups of patients are, indeed, necessary to validate and further explore these preliminary findings.

## Conclusion

This is the first investigation highlighting an important aspect of psychological well-being, that is seldom investigated by clinicians in patients affected by neurological disorders. Based on a multidimensional concept of health-related quality of life, we are in line with other studies (Gündel et al., 2001; Müller et al., 2002; Tan et al., 2005, 2006; Taylor and Gosney, 2011; Bronner et al., 2014) recommending more attention to clinical symptoms not immediately manifest, and adequate psychological support, when necessary.

## Author Contributions

PP conceived and coordinated the study, administered the scales, reviewed the data, and wrote the manuscript. AS was involved in data analysis and interpretation, and also helped with writing. PC was involved in the study concept, interpretation of data and critical revision of the manuscript. RR was involved in data interpretation, critical revision, and writing.

## Conflict of Interest Statement

The authors declare that the research was conducted in the absence of any commercial or financial relationships that could be construed as a potential conflict of interest.
